# 1-Bromo-2,3,6-trichloro-4,5-dimethoxy­benzene

**DOI:** 10.1107/S1600536810002977

**Published:** 2010-01-30

**Authors:** Yang Song, Sean Parkin, Hans-Joachim Lehmler

**Affiliations:** a College of Pharmaceutical Sciences, Southwest University, Chong Qing 400716, People’s Republic of China; b University of Kentucky, Department of Chemistry, Lexington, KY 40506-0055, USA; c The University of Iowa, Department of Occupational and Environmental Health, 100 Oakdale Campus, 124 IREH, Iowa City, IA 52242-5000, USA

## Abstract

The halogen atoms of the title compound, C_8_H_6_BrCl_3_O_2_, are located within the plane of the benzene ring [r.m.s. deviation = 0.036 (11) Å]. The two meth­oxy groups are twisted out of this plane, with dihedral angles of 84.7 (3) and 68.5 (3)°, and point in opposite directions. The structure is disordered by a non-crystallographic twofold rotation which superimposes Cl and Br at two of the halogen sites. The refined occupancies for the major and minor components are 0.517 (2) and 0.483 (2).

## Related literature

For similar structures of halogenated meth­oxy benzenes, see: Iimura *et al.* (1984 ▶); Rissanen *et al.* (1987 ▶, 1988*a* ▶,*b* ▶); Song *et al.* (2008 ▶, 2010 ▶); Telu *et al.* (2008 ▶); Weller & Gerstner (1995 ▶); Wieczorek (1980 ▶). For background to halogenated meth­oxy benzenes, see: Brownlee *et al.* (1993 ▶); Curtis *et al.* (1972 ▶); Pereira *et al.* (2000 ▶); Vlachos *et al.* (2007 ▶); Zhang *et al.* (2006 ▶).
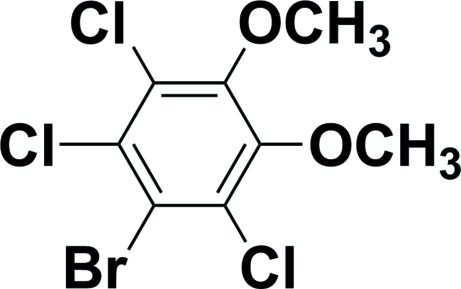


## Experimental

### 

#### Crystal data

C_8_H_6_BrCl_3_O_2_
*M* *_r_* = 320.39
Triclinic, 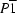

*a* = 7.7885 (7) Å
*b* = 8.8600 (7) Å
*c* = 9.1523 (8) Å
α = 62.256 (3)°
β = 75.358 (4)°
γ = 75.133 (4)°
*V* = 533.64 (8) Å^3^
*Z* = 2
Cu *K*α radiation
μ = 11.94 mm^−1^
*T* = 90 K
0.20 × 0.15 × 0.07 mm

#### Data collection

Bruker X8 Proteum diffractometer
Absorption correction: multi-scan (*SADABS*; Bruker, 2006 ▶) *T*_min_ = 0.193, *T*_max_ = 0.434
6588 measured reflections
1828 independent reflections
1720 reflections with *I* > 2σ(*I*)
*R*_int_ = 0.038

#### Refinement

*R*[*F* ^2^ > 2σ(*F* ^2^)] = 0.038
*wR*(*F* ^2^) = 0.103
*S* = 1.12
1828 reflections
138 parameters
6 restraints
H-atom parameters constrained
Δρ_max_ = 0.35 e Å^−3^
Δρ_min_ = −0.52 e Å^−3^

### 

Data collection: *APEX2* (Bruker, 2006 ▶); cell refinement: *SAINT* (Bruker, 2006 ▶); data reduction: *SAINT*; program(s) used to solve structure: *SHELXS97* (Sheldrick, 2008 ▶); program(s) used to refine structure: *SHELXL97* (Sheldrick, 2008 ▶); molecular graphics: *XP* in *SHELXTL* (Sheldrick, 2008 ▶); software used to prepare material for publication: *SHELXL97* and local procedures.

## Supplementary Material

Crystal structure: contains datablocks I, global. DOI: 10.1107/S1600536810002977/om2317sup1.cif

Structure factors: contains datablocks I. DOI: 10.1107/S1600536810002977/om2317Isup2.hkl

Additional supplementary materials:  crystallographic information; 3D view; checkCIF report

## Figures and Tables

**Fig. 1.**
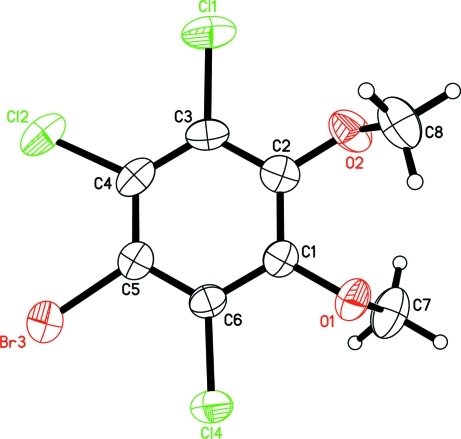
View of the title compound showing the atom-labelling scheme. Displacement ellipsoids are drawn at the 50% probability level.

## supplementary crystallographic information

### Comment

Chlorinated methoxy benzenes are a group of persistent organic pollutants that are associated with off-flavors in water, fish, chicken and wine (Brownlee *et al.*,1993; Curtis *et al.*, 1972; Pereira *et al.*, 2000; Vlachos *et al.*, 2007; Zhang *et al.*, 2006). Their biological properties depend, at least in part, on the conformation of the methoxy group relative to the aromatic ring system. The two methoxy groups of the title compound are twisted out of the plane of the benzene ring system due to the bulky *ortho* chlorine substituents and point in opposite directions. The respective dihedral angles were calculated between the plane of the benzene ring (C1 through C6) and the methoxy group and are 84.7 (3)° (atoms C1,O1,C7) and 68.5 (3)° (atoms C2,O2,C8), respectively. These dihedral angles are in agreement with the dihedral angels observed for other chlorinated methoxy benzenes with two *ortho* substituents (Iimura *et al.*,1984; Rissanen *et al.*, 1987; Rissanen *et al.*, 1988*b*; Telu *et al.*, 2008; Weller & Gerstner, 1995; Wieczorek, 1980). In contrast, the methoxy group of structurally related compound with no or one substituent *ortho* to the methoxy group typically lie within the plane of the benzene ring system (Rissanen *et al.*, 1988*a*; Song *et al.*, 2010).

### Experimental

This title compound was synthesized by chlorination of 1-bromo-3,4-dimethoxy-benzene with HCl/H_2_O_2_ as described previosuly (Song *et al.*, 2008). Crystals suitable for X-ray diffraction were grown by slow evaporation of a saturated solution of the title compound in CHCl_3_.

### Refinement

H atoms were found in difference Fourier maps and subsequently placed in idealized positions with constrained C-H distances of 0.98 Å and U_iso_(H) values set to 1.5U_eq_ of the attached C atom. The structure is disordered by a non-crystallographic 2-fold rotation about an axis that bisects the midpoints of bonds C1—C2 and C4—C5. This disorder superimposes Cl and Br at the halogen sites bonded to C4 and C5. The refined occupancies for the major and minor components are 0.517 (2) and 0.483 (2).  To ensure a physically/chemically sensible model in spite of the disorder, the bond distances for C—Cl were restrained to a refined variable, 1.713 (8). The C—Br bond distance was restrained to the same variable, but multiplied by 1.096, which is the ratio of C—Br:C—Cl for this type of bond.  Six restraints in total were required.  In addition, the displacement parameters of the superimposed atoms were constrained to be the same.

### Crystal data

**Table:**

C_8_H_6_BrCl_3_O_2_	*Z* = 2
*M**_r_* = 320.39	*F*(000) = 312
Triclinic, *P*1	*D*_x_ = 1.994 Mg m^−^^3^
Hall symbol: -P 1	Cu *K*α radiation, λ = 1.54178 Å
*a* = 7.7885 (7) Å	Cell parameters from 5684 reflections
*b* = 8.8600 (7) Å	θ = 5.5–66.1°
*c* = 9.1523 (8) Å	µ = 11.94 mm^−^^1^
α = 62.256 (3)°	*T* = 90 K
β = 75.358 (4)°	Irregular plate, colourless
γ = 75.133 (4)°	0.20 × 0.15 × 0.07 mm
*V* = 533.64 (8) Å^3^	

### Data collection

**Table:**

Bruker X8 Proteum diffractometer	1828 independent reflections
Radiation source: fine-focus rotating anode	1720 reflections with *I* > 2σ(*I*)
graded multilayer optics	*R*_int_ = 0.038
Detector resolution: 18 pixels mm^-1^	θ_max_ = 67.4°, θ_min_ = 5.5°
φ and ω scans	*h* = −9→7
Absorption correction: multi-scan (*SADABS*; Bruker, 2006)	*k* = −10→10
*T*_min_ = 0.193, *T*_max_ = 0.434	*l* = −10→10
6588 measured reflections	

### Refinement

**Table:**

Refinement on *F*^2^	Secondary atom site location: difference Fourier map
Least-squares matrix: full	Hydrogen site location: inferred from neighbouring sites
*R*[*F*^2^ > 2σ(*F*^2^)] = 0.038	H-atom parameters constrained
*wR*(*F*^2^) = 0.103	*w* = 1/[σ^2^(*F*_o_^2^) + (0.0466*P*)^2^ + 0.726*P*] where *P* = (*F*_o_^2^ + 2*F*_c_^2^)/3
*S* = 1.12	(Δ/σ)_max_ = 0.004
1828 reflections	Δρ_max_ = 0.35 e Å^−^^3^
138 parameters	Δρ_min_ = −0.52 e Å^−^^3^
6 restraints	Extinction correction: *SHELXL97* (Sheldrick, 2008), Fc^*^=kFc[1+0.001xFc^2^λ^3^/sin(2θ)]^-1/4^
Primary atom site location: structure-invariant direct methods	Extinction coefficient: 0.0232 (18)

### Special details

**Table:**

Geometry. All e.s.d.'s (except the e.s.d. in the dihedral angle between two l.s. planes) are estimated using the full covariance matrix. The cell e.s.d.'s are taken into account individually in the estimation of e.s.d.'s in distances, angles and torsion angles; correlations between e.s.d.'s in cell parameters are only used when they are defined by crystal symmetry. An approximate (isotropic) treatment of cell e.s.d.'s is used for estimating e.s.d.'s involving l.s. planes.
Refinement. Refinement of *F*^2^ against ALL reflections. The weighted *R*-factor *wR* and goodness of fit *S* are based on *F*^2^, conventional *R*-factors *R* are based on *F*, with *F* set to zero for negative *F*^2^. The threshold expression of *F*^2^ > 2σ(*F*^2^) is used only for calculating *R*-factors(gt) *etc*. and is not relevant to the choice of reflections for refinement. *R*-factors based on *F*^2^ are statistically about twice as large as those based on *F*, and *R*- factors based on ALL data will be even larger.

### Fractional atomic coordinates and isotropic or equivalent isotropic displacement parameters (Å^2^)

**Table:**

	*x*	*y*	*z*	*U*_iso_*/*U*_eq_	Occ. (<1)
C1	0.2391 (4)	0.6704 (4)	0.7165 (4)	0.0318 (6)	
C2	0.2512 (4)	0.7913 (5)	0.7681 (4)	0.0360 (7)	
C3	0.2598 (4)	0.9603 (4)	0.6519 (4)	0.0356 (7)	
C4	0.2536 (4)	1.0107 (4)	0.4851 (4)	0.0366 (7)	
C5	0.2393 (4)	0.8899 (4)	0.4345 (4)	0.0350 (7)	
C6	0.2314 (4)	0.7212 (4)	0.5504 (4)	0.0329 (7)	
O1	0.2295 (3)	0.5051 (3)	0.8291 (3)	0.0418 (6)	
C7	0.3984 (6)	0.3991 (5)	0.8622 (6)	0.0592 (11)	
H7A	0.4694	0.3956	0.7585	0.089*	
H7B	0.3800	0.2818	0.9444	0.089*	
H7C	0.4627	0.4464	0.9066	0.089*	
O2	0.2646 (3)	0.7442 (4)	0.9285 (3)	0.0486 (6)	
C8	0.1070 (5)	0.6975 (6)	1.0458 (5)	0.0558 (10)	
H8A	0.0096	0.7968	1.0188	0.084*	
H8B	0.1309	0.6619	1.1584	0.084*	
H8C	0.0717	0.6014	1.0412	0.084*	
Cl1	0.27710 (13)	1.10613 (13)	0.71869 (14)	0.0567 (3)	
Cl2	0.265 (3)	1.2206 (17)	0.326 (2)	0.0539 (7)	0.483 (3)
Br3	0.2388 (9)	0.9427 (6)	0.2147 (7)	0.0495 (7)	0.483 (3)
Br2	0.2599 (11)	1.2383 (6)	0.3376 (8)	0.0539 (7)	0.517 (3)
Cl3	0.228 (2)	0.9701 (14)	0.2211 (15)	0.0495 (7)	0.517 (3)
Cl4	0.20968 (13)	0.56783 (11)	0.49546 (11)	0.0485 (3)	

### Atomic displacement parameters (Å^2^)

**Table:**

	*U*^11^	*U*^22^	*U*^33^	*U*^12^	*U*^13^	*U*^23^
C1	0.0236 (14)	0.0338 (16)	0.0354 (16)	−0.0092 (12)	−0.0006 (11)	−0.0123 (13)
C2	0.0257 (15)	0.0442 (18)	0.0406 (17)	−0.0106 (13)	0.0000 (12)	−0.0203 (15)
C3	0.0255 (15)	0.0388 (17)	0.0513 (19)	−0.0071 (12)	−0.0013 (13)	−0.0280 (15)
C4	0.0242 (15)	0.0279 (15)	0.0501 (19)	−0.0039 (12)	0.0005 (13)	−0.0136 (14)
C5	0.0269 (15)	0.0348 (16)	0.0392 (16)	−0.0046 (12)	−0.0039 (12)	−0.0133 (14)
C6	0.0291 (15)	0.0323 (16)	0.0387 (16)	−0.0080 (12)	−0.0021 (12)	−0.0163 (13)
O1	0.0424 (13)	0.0343 (12)	0.0402 (12)	−0.0132 (10)	−0.0033 (10)	−0.0072 (10)
C7	0.054 (2)	0.040 (2)	0.060 (2)	0.0007 (17)	−0.0095 (19)	−0.0062 (18)
O2	0.0456 (14)	0.0687 (17)	0.0418 (13)	−0.0189 (12)	−0.0064 (10)	−0.0275 (13)
C8	0.050 (2)	0.076 (3)	0.0380 (19)	−0.009 (2)	−0.0024 (16)	−0.0245 (19)
Cl1	0.0559 (6)	0.0536 (6)	0.0797 (7)	−0.0177 (4)	0.0007 (5)	−0.0454 (5)
Cl2	0.0526 (6)	0.0296 (11)	0.0692 (11)	−0.0075 (10)	−0.0009 (7)	−0.0165 (7)
Br3	0.0579 (9)	0.0403 (16)	0.0456 (6)	−0.0037 (12)	−0.0149 (5)	−0.0133 (8)
Br2	0.0526 (6)	0.0296 (11)	0.0692 (11)	−0.0075 (10)	−0.0009 (7)	−0.0165 (7)
Cl3	0.0579 (9)	0.0403 (16)	0.0456 (6)	−0.0037 (12)	−0.0149 (5)	−0.0133 (8)
Cl4	0.0670 (6)	0.0407 (5)	0.0471 (5)	−0.0185 (4)	−0.0078 (4)	−0.0221 (4)

### Geometric parameters (Å, °)

**Table:**

C1—O1	1.351 (4)	C5—Cl3	1.758 (12)
C1—C6	1.383 (5)	C5—Br3	1.842 (6)
C1—C2	1.388 (5)	C6—Cl4	1.710 (3)
C2—O2	1.350 (4)	O1—C7	1.417 (5)
C2—C3	1.382 (5)	C7—H7A	0.9800
C3—C4	1.387 (5)	C7—H7B	0.9800
C3—Cl1	1.711 (3)	C7—H7C	0.9800
C4—C5	1.386 (5)	O2—C8	1.418 (5)
C4—Cl2	1.756 (15)	C8—H8A	0.9800
C4—Br2	1.837 (6)	C8—H8B	0.9800
C5—C6	1.379 (5)	C8—H8C	0.9800
			
O1—C1—C6	120.3 (3)	C4—C5—Br3	122.6 (3)
O1—C1—C2	120.0 (3)	C5—C6—C1	120.8 (3)
C6—C1—C2	119.7 (3)	C5—C6—Cl4	121.6 (3)
O2—C2—C3	119.5 (3)	C1—C6—Cl4	117.6 (2)
O2—C2—C1	120.9 (3)	C1—O1—C7	114.5 (3)
C3—C2—C1	119.5 (3)	O1—C7—H7A	109.5
C2—C3—C4	120.7 (3)	O1—C7—H7B	109.5
C2—C3—Cl1	118.5 (3)	H7A—C7—H7B	109.5
C4—C3—Cl1	120.8 (3)	O1—C7—H7C	109.5
C5—C4—C3	119.5 (3)	H7A—C7—H7C	109.5
C5—C4—Cl2	115.5 (6)	H7B—C7—H7C	109.5
C3—C4—Cl2	125.0 (7)	C2—O2—C8	115.4 (3)
C5—C4—Br2	121.8 (3)	O2—C8—H8A	109.5
C3—C4—Br2	118.7 (3)	O2—C8—H8B	109.5
C6—C5—C4	119.7 (3)	H8A—C8—H8B	109.5
C6—C5—Cl3	125.0 (5)	O2—C8—H8C	109.5
C4—C5—Cl3	115.3 (4)	H8A—C8—H8C	109.5
C6—C5—Br3	117.6 (3)	H8B—C8—H8C	109.5
			
O1—C1—C2—O2	4.0 (5)	Br2—C4—C5—Cl3	0.1 (7)
C6—C1—C2—O2	−177.9 (3)	C3—C4—C5—Br3	−177.6 (3)
O1—C1—C2—C3	−179.6 (3)	Cl2—C4—C5—Br3	1.8 (9)
C6—C1—C2—C3	−1.5 (5)	Br2—C4—C5—Br3	3.7 (5)
O2—C2—C3—C4	177.4 (3)	C4—C5—C6—C1	−0.6 (5)
C1—C2—C3—C4	1.0 (5)	Cl3—C5—C6—C1	−179.2 (7)
O2—C2—C3—Cl1	−2.9 (4)	Br3—C5—C6—C1	177.2 (3)
C1—C2—C3—Cl1	−179.3 (2)	C4—C5—C6—Cl4	178.8 (2)
C2—C3—C4—C5	−0.3 (5)	Cl3—C5—C6—Cl4	0.2 (8)
Cl1—C3—C4—C5	−179.9 (2)	Br3—C5—C6—Cl4	−3.4 (4)
C2—C3—C4—Cl2	−179.6 (9)	O1—C1—C6—C5	179.4 (3)
Cl1—C3—C4—Cl2	0.7 (10)	C2—C1—C6—C5	1.3 (5)
C2—C3—C4—Br2	178.5 (4)	O1—C1—C6—Cl4	0.0 (4)
Cl1—C3—C4—Br2	−1.2 (5)	C2—C1—C6—Cl4	−178.1 (2)
C3—C4—C5—C6	0.0 (5)	C6—C1—O1—C7	96.2 (4)
Cl2—C4—C5—C6	179.4 (8)	C2—C1—O1—C7	−85.7 (4)
Br2—C4—C5—C6	−178.7 (4)	C3—C2—O2—C8	113.2 (4)
C3—C4—C5—Cl3	178.8 (6)	C1—C2—O2—C8	−70.5 (4)
Cl2—C4—C5—Cl3	−1.8 (10)		
